# Clinical Management of Patients With B-Cell Depletion Agents to Treat or Prevent Prolonged and Severe SARS-COV-2 Infection: Defining a Treatment Pathway

**DOI:** 10.3389/fimmu.2022.911339

**Published:** 2022-05-27

**Authors:** Alessandra D’Abramo, Serena Vita, Gaetano Maffongelli, Alessia Beccacece, Chiara Agrati, Eleonora Cimini, Francesca Colavita, Maria Letizia Giancola, Alessandro Cavasio, Emanuele Nicastri, Spallanzani COVID-19 Case Investigation Team, Angela Corpolongo

**Affiliations:** ^1^ National Institute for Infectious Diseases “Lazzaro Spallanzani” (IRCCS), Rome, Italy; ^2^ Clinical Infectious Diseases, Department of System Medicine, Tor Vergata University, Rome, Italy

**Keywords:** immunosuppressed patients, COVID-19, passive immunotherapy, anti-CD20 agent, B-cells depletion, convalescent plasma, anti-SARS-CoV-2 monoclonal antibody

## Abstract

**Introduction:**

Immunocompromised patients with B-cell depletion agents are at risk for persistence and/or severe SARS-COV-2 infection. We describe a case series of 21 COVID-19 patients under B cell depletion therapy, mostly treated with a combined therapy based on intravenous remdesevir (RDV) and steroid associated with SARS-CoV-2 monoclonal antibodies against Spike glycoprotein and/or hyper-immune convalescent plasma.

**Methods:**

This is a single-center longitudinal study. We retrospectively enrolled a total number of 21 B-cell depleted consecutive hospitalized patients with COVID-19 at the Lazzaro Spallanzani National Institute for Infectious Diseases, Rome, Italy, from November 2020 to December 2021. Demographic characteristics, medical history, clinical presentation, treatment, adverse drug reactions, and clinical and virological outcome were collected for all patients. In a subgroup, we explore immune T cells activation, T cells specific anti-SARS-COV-2 response, and neutralizing antibodies.

**Results:**

Twenty-one inpatients with B-cell depletion and SARS-COV-2 infection were enrolled. A median of 1 B cells/mm^3^ was detected. Eighteen patients presented hypogammaglobulinemia. All patients presented interstitial pneumonia treated with intravenous RDV and steroids. Sixteen patients were treated with monoclonal antibodies against SARS-CoV-2 Spike protein, four patients were treated with SARS-CoV-2 hyper-immune convalescent plasma infusion, and three patients received both treatments. A variable kinetic of T cell activation returning to normal levels at Day 30 after immunotherapy infusion was observed. All treated patients recovered.

**Conclusion:**

In COVID-19 immunosuppressed subjects, it is mandatory to establish a prompt, effective, and combined multi-target therapy including oxygen, antiviral, steroid, and antibody-based therapeutics, tailored to the patient’s clinical needs.

## Introduction

Immunocompromised patients with autoimmune, onco-hematologic, and/or neurologic disorders, with B cell depletion and negative serologic evidence against SARS-CoV-2 after both natural infection and/or SARS-CoV-2 vaccination are at higher risk of severe and/or prolonged COVID-19.

Among these patients anti-CD20 antibody-based B cell-depleting strategies such as rituximab are widely used (1). Upon antigen exposure, B cells can form memory cells or differentiate into plasmablasts and plasma cells. Memory B cells are precursors to antibody-secreting cells, and in addition they can function as professional antigen presenting cells, especially in the context of interactions with T cells that recognize the same antigenic target (2). Treatment with rituximab results in complete B cell-depletion within 72 h from administration, with an estimated recovery timing in 6-9 months after the completion of therapy, and with a return to normal levels observed after 9-12 months (3). These long-lasting therapies are associated with an increased risk of infections such as tuberculosis, hepatitis B virus, herpes virus reactivation, and SARS-CoV-2 infection (4).

The ongoing pandemic is a serious issue for patients treated with anti-CD20 monoclonal or similar biological agents with a COVID-19 mortality rate as high as 60% (5). Indeed, immunocompromised patients could have clinical and virological evidence of persistent SARS-CoV-2 infection of more than 21 days’ duration and/or more than 2 episodes of acute respiratory syndrome (6, 7). In this setting, it is important to identify and approach these patients from different perspectives in terms of clinical, diagnostic, and therapeutic options. In the last months, scientific evidence on clinical approaches based on passive immunotherapy has been successfully reported (8). Passive immunotherapy such as hyperimmune convalescent plasma and/or monoclonal antibodies (MoAbs) against SARS-CoV-2 infection represent a source of exogenous specific antibodies in immunocompromised patients with primary or secondary humoral disorders (9–11). To date, there are no available robust data to provide evidence-based protocols on the management of immunosuppressed patients. Here, we describe a case series of 21 COVID-19 patients, under B cell depletion therapy, of whom 20 successfully treated with SARS-CoV-2 MoAbs against Spike glycoprotein and/or hyper-immune convalescent plasma.

## Methods

In this single-center cross-sectional study, we retrospectively enrolled a total number of 21 immunosuppressed patients consecutively hospitalized with prolonged or relapsing COVID-19 (with >21 days’ duration and/or >2 episodes of clinical illness) at the Lazzaro Spallanzani National Institute for Infectious Diseases, Rome, Italy (INMI Spallanzani), from November 2020 to December 2021. Demographic characteristics, medical history, clinical presentation, treatment, adverse drug reactions, and clinical outcome (survival/death) at Day 28 post-treatment were collected for all patients from the clinical record.

In a subgroup of 11 patients the expression of CD38 activation marker on CD4 and CD8 T cells was evaluated by flow cytometry at baseline (T0), after 3 (T3), 7 (T7), 14 (T14), and 30 days (T30) of MoAbs and hyperimmune convalescent plasma. The following gating strategy was used to identify T cells population: CD4+ and CD8+ T cells were identified in CD3+ T cells, gated in SSC-A/CD45+ cells, and within SSC-H/SSC-A in FSC singlets gate.

Spike- and Nucleocapside-specific T cells were quantified by IFN-γ Elispot assay, following the kit’s instruction. Positive control peripheral blood mononuclear cells (PBMCs) were stimulated with phytohemagglutinin (PHA) (data not shown). As a negative control, the spontaneous interferon (IFN)-γ release by unstimulated PBMC was quantified.

Real-time reverse transcription polymerase chain reaction (RT-PCR) was performed according to the laboratory workflow using different platforms: DiaSorin Simplexa^®^ COVID-19 Direct platform, Abbott m2000 RealTime System, and the Cobas^®^ SARS-CoV-2 Test on the fully automated cobas^®^ 6800 Systems. Viral characterization was performed on nasopharyngeal swab (NPS) samples collected, when possible, by Next-Generation Sequencing (NGS) on Ion Torrent Platform using Ion AmpliSeq SARS-CoV-2 Research Panel, following the manufacturer’s instructions

SARS-COV-2 serology was performed by an enzyme-linked immunosorbent assay (ELISA) detecting anti-SARS-CoV-2 IgG, IgM, and IgA (ENZY-WELL SARS-CoV-2 ELISA on SkyLab platform; DIESSE), or by two chemiluminescence microparticle assays (CMIA) detecting anti-nucleoprotein (anti-N) IgG and anti-Spike/RBD IgG (ARCHITECT SARS-CoV-2 IgG, and ARCHITECT SARS-CoV-2 IgG II Quantitative, on ARCHITECT^®^ i2000sr; Abbott Laboratories, Wiesbaden, Germany, respectively). According to the manufacturer’s instructions, index values S/CO ≥ 1.1 are considered positive for ELISA, while for the two CMIA, Index >1.4 and Binding Antibody Units (BAU)/mL ≥7.1 are considered positive for anti-N and anti-Spike/RBD IgG, respectively.

Neutralizing antibodies were assessed by micro-neutralization assay (MNA) using live SARS-CoV-2. Briefly, seven twofold serial dilutions (starting dilution 1:10) of heat-inactivated serum samples (56°C for 30 min) were titrated in duplicate, mixed with 100 TCID50 SARS-CoV-2 and incubated at 37°C for 30 min. Subsequently, virus-serum mixtures were added to sub-confluent Vero E6 cells seeded in 96-well microplates and incubated at 37°C, 5% CO_2_. After 48 h, microplates were observed by light microscope for the presence of cytopathic effect (CPE). Neutralization titers were expressed as the reciprocal of the highest serum dilution inhibiting at least 90% (MNA90) of CPE.

## Results

From November 2020 to December 2021, 21 patients with B-cells depletion and SARS-COV-2 infection were enrolled: 12 men with a median age of 65 (InterQuartile Range – IQR, 54-71.5) years old. **Tables 1**, **2** show patients’ clinical and laboratory features (data are reported in chronological order of admission). Among 21 patients, 15 patients had a hematological disorder, 2 patients had a multiple sclerosis, and 4 patients had an autoimmune disease (2 Wegener granulomatosis, 1 psoriatic arthritis, and 1 rheumatoid arthritis). All study population had a history of immunosuppression (median of 1 B cells/mm3, IQR 0-2 cells): 16 patients for a previous treatment with anti-CD20 agents, 2 patients for a previous treatment with anti-CD38 agents, and 2 for previous standard chemotherapy. Residual hypogammaglobulinemia was observed in 18 patients. Of them, 7 subjects were COVID-19 vaccinated. Viral characterization was performed on nasopharyngeal swab samples collected from 4 of the 21 patients. Two patients tested positive for the Alpha variant, 1 patient for a B.1.177.33 lineage (GV clade) and a B.1.177 lineage (BGV), respectively.

**Table 1:** Study population in chronological order of admission: clinical features.

Pt	Sex	Age	Ongoing IS therapy	Days from last IS administration	Diseases	Days to hospitalization from symptom onset	Days to hospitalization from first NPS	MoAbs	Hyperimmuneplasma	VS	Lenght of stay	Days to PCR negativization	Clinical Outcome
1	F	54	Ocrelizumab	105	MS	20	21	CAS+IMD	No	NONE	51	55	RECOVERY
2	M	54	Rituximab	163	NHL	31	17	CAS+IMD	No	NIV	32	68	RECOVERY
3	M	39	Rituximab	>12 y	NHL	54	63	NO	Yes	NIV	92	85	RECOVERY
4	M	66	Obinutumab	NA	NHL	52	100	CAS+IMD	Yes	VM	19	168	RECOVERY
5	M	65	Rituximab	60	NHL	33	33	NO	Yes	VM	19	50	RECOVERY
6	M	67	Rituximab	106	NHL	3	114	NO	Yes	VM	30	123	RECOVERY
7	M	59	Rituximab	150	NHL	120	6	NO	Yes	VM	15	43	RECOVERY
8	F	24	Rituximab	76	WG	78	6	BAM+ETE	No	VM	11	90	RECOVERY
9	F	25	Ocrelizumab	13	MS	12	78	CAS+IMD	No	VM	19	38	RECOVERY
10	F	66	Rituximab	180	NHL	7	7	CAS+IMD	No	c-PAP	26	76	RECOVERY
11	F	77	Rituximab	217	NHL	10	8	CAS+IMD	Yes	NIV	51	90	RECOVERY
12	M	35	Rituximab	126	PS	46	47	CAS+IMD	No	c-PAP	62	61	RECOVERY
13	M	54	Rituximab	11 y	NHL	120	119	BAM+ETE	Yes	NIV	6	119	RECOVERY
14	F	68	Rituximab	66	CLL	36	36	CAS+IMD	No	c-PAP	44	60	RECOVERY
15	M	68	Rituximab	NA	NHL	13	10	NO	No	VM	30	NA	DEATH
16	M	73	Daratumumab	NA	MM	2	11	CAS+IMD	No	VM	38	36	RECOVERY
17	F	76	Rituximab	NA	RA	9	9	CAS+IMD	No	VM	11	37	RECOVERY
18	M	83	None	>20 y	NHL	9	9	CAS+IMD	No	VM	16	31	RECOVERY
19	M	63	None	2 y	NHL	41	2	CAS+IMD	No	VM	35	36	RECOVERY
20	F	77	Daratumumab	30	MM	3	2	CAS+IMD	No	c-PAP	30	39	RECOVERY
21	F	56	Rituximab	14	WG	4	4	CAS+IMD	No	VM	17	28	RECOVERY

IS, immunosoppressive; NPS, nasopharyngeal swab; MoAbs, monoclonal antibodies; CAS, casirivimab, IMD, imdevimab; BAM, bamlanivimab; ETE, etesevimab; VS, ventilatory support; MS, multiple sclerosis; NHL, non-Hodgkin’s lymphoma; PS, psoriatic arthritis; WG, Wegener granulomatosis; MM, multiple myeloma; VM, venturi mask; c-PAP, continuous positive air pressure; NIV, non-invasive ventilation; IOT, orotracheal intubation; NA, not available.

**Table 2:** Immunological features in the study population.

Pt	Hypogammaglobulinemia	CD20/mm^3	SARS-CoV-2 vaccination	Pre-immunotherapy			Post-Immunotherapy		
				IgM/IgA/IgG	Neutralizing Abs	Anti-Spike/RBD IgG	IgM/IgA/IgG	Neutralizing Abs	Anti-Spike/RBD IgG
1	Yes	1	None	+/-/-	NA	NEG	-/-/+	>1:640	8897.7
2	Yes	1	None	-/-/-	NA	NEG	-/+/+	1:160	2961.1
3	Yes	0	None	-/-/-	<1:10	NEG	-/-/-	<1:10	181
4	No	1	None	-/-/-	<1:10	NEG	-/-/+	1:80	1159
5	Yes	0	None	-/-/-	<1:10	NEG	-/-/-	<1:10	67
6	Yes	1	None	-/-/-	<1:10	NEG	-/-/-	<1:10	197
7	Yes	1	II Doses	-/-/-	<1:10	NEG	-/+/+	NA	NA
8	Yes	2	None	+/-/-	<1:10	NEG	-/-/+	>1:640	>11360
9	No	0	None	-/-/+	NA	NA	-/-/+	>1:640	>11360
10	Yes	2	II Doses	-/-/-	<1:10	NEG	-/-/+	NA	NA
11	Yes	2	I Dose	-/-/-	<1:10	NEG	-/-/+	NA	NA
12	Yes	1	None	-/-/-	<1:10	13.7	-/-/+	NA	NA
13	Yes	24	None	-/-/-	<1:10	NEG	-/-/+	1:20	420.9
14	Yes	0	II Doses	-/-/-	NA	NA	-/-/+	NA	NA
15	Yes	0	None	NA	NA	NA	NA	NA	NA
16	Yes	0	None	-/-/-	NA	NEG	-/-/+	NA	>11360
17	NA	0	II Doses	-/-/-	NA	NEG	-/-/+	NA	NA
18	Yes	3	II Doses	-/-/-	<1:10	NEG	-/-/+	>1:640	>11360
19	Yes	24	None	-/-/-	NA	NA	-/-/+	NA	NA
20	Yes	19	III Doses	-/-/-	<1:10	NEG	-/-/+	NA	>11360
21	Yes	2	None	-/-/-	NA	NA	-/-/+	NA	NA

Ig, immunoglobulins; Abs, antibodies; NA, not available. Anti-Spike/RBD IgG are expressed as Binding Antibody Units (BAU)/mL, values ≥7.1 are considered positive; neutralizing titers >1:10 are considered positive.

**Figure 1:**
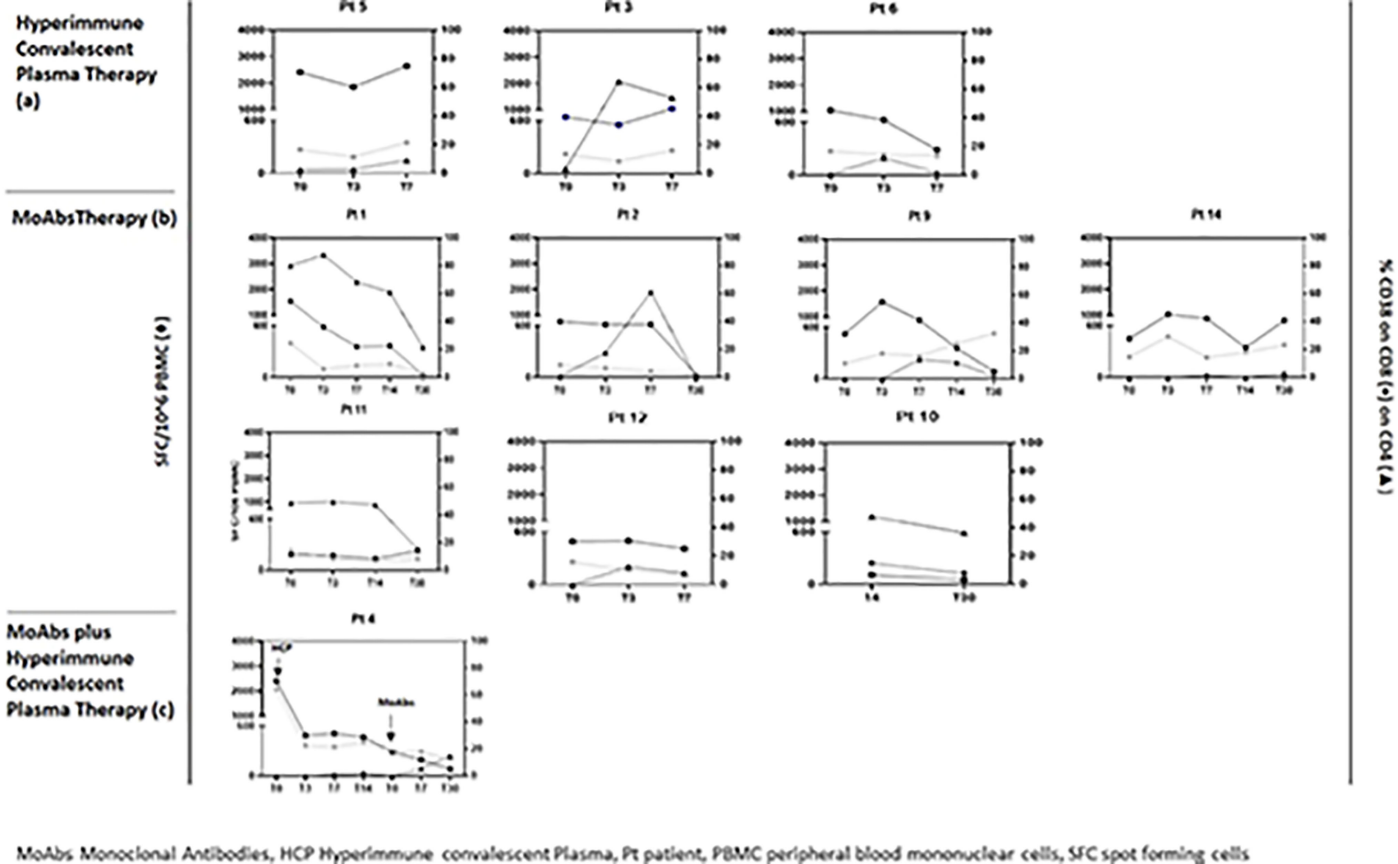
Impact of hyperimmune convalescent plasma **(A)** anti-SARS-CoV-2 monoclonal antiboby **(B)** and hyperimmune convalescent plasma plus anti-SARS-CoV-2 monoclonal antibody **(C)** therapy on immune parameters and specific T cell response in peripheral blood before and after treatment.

Nine patients had a prolonged COVID-19 infection; the remaining 12 patients had a primary infection. All patients presented interstitial pneumonia treated with intravenous remdesivir (RDV) and steroids. Sixteen patients were treated with MoAbs against SARS-CoV-2 Spike glycoprotein (casirivimab/imdevimab in 14 cases and bamlanivimab/etesevimab in 2 cases), 4 patients were treated with SARS-CoV-2 hyper-immune convalescent plasma infusion (1:320 neutralizing Abs against SARS-CoV-2 Spike glycoprotein), and 3 patients received both treatments. One patient died, he refused both treatments. The median time to hospitalization from the first positive nasopharyngeal swab was 11 (IQR 6.25-59) days, the median SARS-CoV-2 viral shedding was 57.5 (IQR, 36.5-90) days, and the median length of hospitalization was 30 days (IQR 16.25-42.5). Stratifying patients according to treatment, a decreasing trend of length of stay (LS), and time to negative SARS-CoV-2 molecular NPS in MoAbs vs. hyperimmune convalescent plasma treated patients was observed (28.6 vs. 39 days for LS and 50.3 vs. 75.2 days for negative NPS, respectively). Oxygen supports varied according to patient clinical severity but no differences in terms of antiviral and MoAb therapies and of clinical outcome were reported. CD4 and CD8 T cell activation (CD38^+^) was evaluated in 11 patients: 7 patients were treated with MoAbs, 3 patients with plasma, and 1 patient with both regimens. A variable kinetic of T cell activation returning to normal levels at Day 30 after immunotherapy infusion was shown. Interestingly, in the patient sequentially treated first, with plasma infusion and then with MoAb therapy, a significant early reduction of T cell activation was reported. Therefore, all patients showed a positive Spike-specific T cell response that reached a peak between Day 7 and 14 after treatment and then slowed down. In contrast, the patient treated with both therapeutics had a delayed specific T cell response after MoAb infusion only (**Figure 1**).

## Discussion

Patients receiving B-cell lymphocyte depletion agents have been shown to be particularly vulnerable to COVID-19, having four times higher odds of COVID-19-related case fatality rate compared with patients on other immunosuppressive medication (12). Indeed, this group of patients usually has a prolonged viral clearance in the respiratory tract with more than 21 days duration, several episodes of acute respiratory infectious syndrome and no SARS-CoV-2 Spike glycoprotein or N protein seroconversion even at Day 20 from symptom onset (7, 12–15). Here, we described the clinical, virological, and immunological pictures of 20 patients with B-cell depletion and SARS-CoV-2 pneumonia ranging from mild to severe, of them 9 had a prolonged infection.

All subjects were treated with intravenous RDV plus systemic corticosteroid associated with passive immunotherapy with SARS-CoV-2 MoAbs and/or hyperimmune convalescent plasma. This therapeutic approach was adopted in all patients even in those with primary SARS-COV-2 infection because of the higher risk of prolonged or severe COVID-19. Although RDV and MoAb use is recommended in COVID-19 patients in the early phase of SARS-CoV-2 infection, RDV was administered after Day 10 in 65% of cases, and MoAbs after Day 7 in 80% of cases (16).

Randomized controlled clinical trials on hyperimmune convalescent plasma have shown controversial results but clinical benefit has been recently demonstrated when used early within 72 h from symptoms’ onset and with high titers of neutralizing antibodies (17). Nevertheless, in our cohort of immune suppressed patients undergoing anti-CD20 biological agents, antivirals and passive immunotherapy have been used later than in the early phase of the infection as they have been studied, suggesting an additional clinical benefit of this combination strategy even in reducing long lasting viral replication. The use of systemic steroids was balanced in all patients, regardless of their immunosuppressed status. In the subgroup of patients with immunological data, T cells activation levels returned to normal levels at Day 30 from immunotherapy, and it was more frequently observed in MoAb-treated than in plasma-treated patients. In one patient first treated with plasma, T cell activation was reduced after MoAb infusion only. Despite the limit of the small sample size of our cohort, a decreasing trend of both length of stay and time to negative molecular NPS in MoAb-treated vs. plasma-treated patients was observed.

Passive immunotherapy (convalescent plasma and/or MoAbs) against SARS-CoV-2 infection represents a main therapeutic option as a source of exogenous specific antibodies in immunocompromised patients with primary or secondary humoral disorders (18). Although to date there is no therapeutic consensus in immunocompromised patients, anecdotal case series of clinical success with combined anti SARS-COV-2 therapy (10, 11) are consistent with our preliminary data. Passive immunotherapy associated with remdesivir, systemic steroid, and oxygen therapy is a successful and safe strategy in our immunosuppressed COVID-19 cohort in terms of reducing viral load, immunoactivation, and case fatality.

Rapid viral clearance, shorter duration of hospitalization, and full recovery were observed in patients treated in 2021 compared to 2020 when this therapeutic approach was early adopted as a combined drug strategy immediately after hospital admission to prevent SARS-COV-2 persistence.

Limitations to MoAb use are related to its limited availability, currently almost exclusive of patients from high-income countries, to its high cost, to the time required for regulatory approval for routine clinical use, and to drug procurement difficulties during Covid-19 epidemic peaks. Finally, susceptibility to MoAb therapies may be reduced because of changes in circulating variants of concern (19).

A likely alternative to MoAb is the hyperimmune convalescent plasma, which is available in low- and middle-income countries without patent restrictions and at relatively low cost. Hyperimmune convalescent plasma provides a diverse mixture of antibodies with different specificities and functions and should be less vulnerable to the emergence of antibody resistance and viral variants (19). Finally, underlying medical conditions (onco-hematologic malignancies, neurologic and autoimmune disorders), disease activity status, and concomitant/previous treatments that affect immune cell distribution and function are all factors to be considered in the choice of specific treatment for selected categories of COVID-19 patients. In COVID-19 immunosuppressed subjects, it is mandatory to establish a prompt, effective, safe, and combined multi-target therapy including oxygen, antiviral, and steroid and antibody-based therapeutics, tailored to the patient’s clinical needs.

## Data Availability Statement

The original contributions presented in the study are included in the article/supplementary material. Further inquiries can be directed to the corresponding author.

## Ethics Statement

Ethical review and approval was not required for the study on human participants in accordance with the local legislation and institutional requirements. The patients/participants provided their written informed consent to participate in this study.

## Spallanzani COVID-19 Case Investigation Team

Angela Corpolongo, Laura Scorzolini, Tommaso Ascoli Bartoli, Claudia Palazzolo, Nazario Bevilacqua, Andrea Mariano, Neva Braccialarghe, Silvia Rosati, Mattia Albanese, Domenico Benvenuto, Giulia Matusali, Massimo Francalancia, Aurora Bettini, Concetta Castilletti, Stefania Notari, Anna Rosa Garbuglia, Silvia Meschi.

## Author Contributions

Conceptualization, AD’A, SV, GM, and EN; Data curation, AB and AC; Funding acquisition, EN; Investigation, AD’A, SV, GM, and ML; Experiments, EC, FC, and CA; Supervision, EN and CA; Validation, EN; Writing—original draft, AD’A, SV, and EN; Writing—review and editing, CA, FC, EC, ML, and EN. All authors contributed to the article and approved the submitted version.

## Funding

This work was supported by Line1 Ricerca Corrente “Studio dei patogeni ad alto impatto sociale: emergent, da importazione, multiresistenti, negletti” funded by the Italian Ministry of Health.

## Conflict of Interest

The authors declare that the research was conducted in the absence of any commercial or financial relationships that could be construed as a potential conflict of interest.

## Publisher’s Note

All claims expressed in this article are solely those of the authors and do not necessarily represent those of their affiliated organizations, or those of the publisher, the editors and the reviewers. Any product that may be evaluated in this article, or claim that may be made by its manufacturer, is not guaranteed or endorsed by the publisher.
